# Acute Intracoronary Thrombosis in a Normal Coronary Artery Following Coronary Angiography: Thromboaspiration Using a Guide Catheter

**DOI:** 10.4103/1995-705X.73218

**Published:** 2010

**Authors:** Prashanth Panduranga, Abdullah Amour Riyami

**Affiliations:** Department of Cardiology, Royal Hospital, Muscat, Sultanate of Oman

**Keywords:** Coronary vasospasm, intracoronary thrombus, thromboaspiration

## Abstract

A 60-year-old female presented with anterolateral non-ST elevation myocardial infarction and her coronary angiogram revealed severe left system coronary artery disease with a normal right coronary artery. Following coronary angiogram, she developed acute inferior wall and right ventricular ST elevation myocardial infarction with complete atrioventricular block and cardiogenic shock. Repeat coronary angiogram showed large proximal right coronary thrombus causing subtotal occlusion that was successfully aspirated using a guide catheter. The possible causes for intracoronary thrombosis following coronary angiography are discussed here.

## CASE PRESENTATION

**Figure 1:**
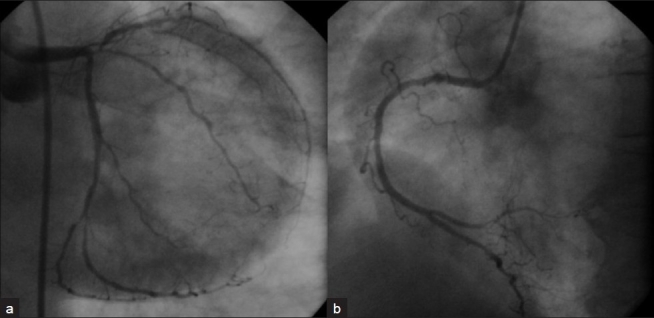
Left coronary angiogram (a) showing critical stenosis involving the left system and right coronary angiogram (b) showing a normal proximal right coronary artery

**Figure 2:**
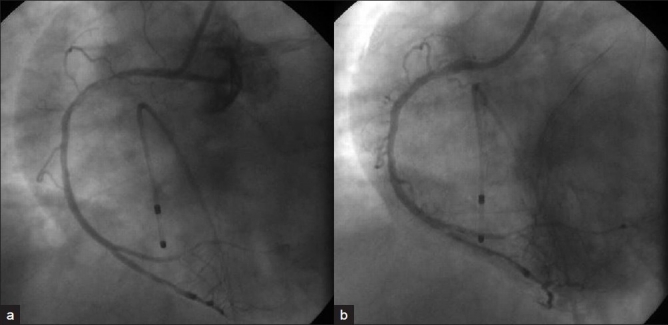
(a) Right coronary angiogram showing proximal subtotal occlusion with thrombus following initial coronary angiogram. (b) Right coronary angiogram showing relief of occlusion after thrombus aspiration with TIMI 3 flow

**Figure 3:**
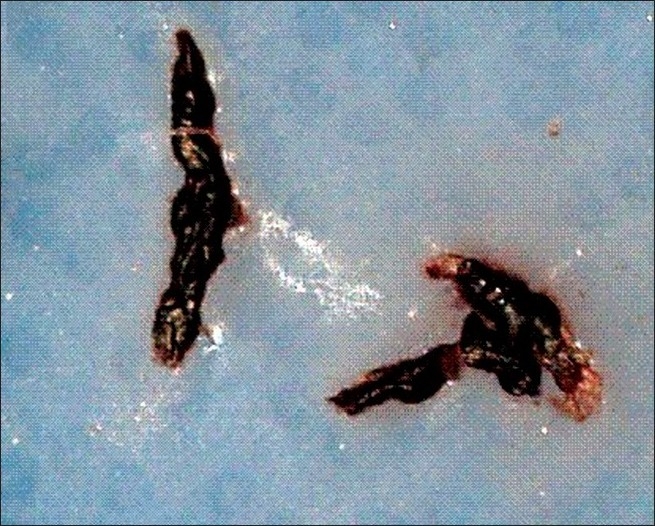
Two large clots removed from the right coronary artery through the guide catheter

A60-year-old female with no coronary risk factors presented to our hospital with chest pain. ECG showed sinus rhythm with 2- to 3-mm ST depression in chest leads and troponin T was positive. Her echocardiogram showed severe anterolateral hypokinesia with ejection fraction of 20% and no intracardiac thrombus. She was treated with aspirin, clopidogrel, statin, intravenous nitroglycerin, and unfractionated heparin infusion. She underwent urgent coronary angiogram from right femoral approach using 6 French Judkins diagnostic catheters, which revealed tight 99% ostial and 95% mid-left anterior descending artery stenosis, and 99% ostial ramus intermediate artery stenosis along with mid and distal tight stenosis of the left circumflex artery [Figure 1a]. The right coronary artery (RCA) was normal [Figure 1b]. The procedure was uneventful. She was scheduled for urgent coronary artery bypass surgery. Fifteen minutes post-coronary angiogram, the patient developed severe chest pain with 5 mm ST elevation in leads II, III, AVF, and V4R along with complete atrioventricular block and cardiogenic shock. Her systolic blood pressure was 60 mmHg with a heart rate of 40 beats per minute. She was started on inotropes and taken for emergency percutaneous coronary intervention (PCI). After temporary wire and intra-aortic balloon pump insertion, left coronary injection showed the same findings as before, but the RCA injection using the 6 French JR guide catheter showed subtotal occlusion of the proximal RCA with a long luminal filling defect suggestive of thrombus without flow compromise [Figure 2a]. There was no response to intracoronary nitroglycerin. The occlusion was crossed with a coronary guide wire. As there was a large clot burden, first we planned to aspirate the thrombus. The aspiration catheter was not available with us; hence, after deep intubation of the JR guide catheter, we manually aspirated two large clots [Figure 3] with immediate relief of the occlusion [Figure 2b] and no significant stenosis or dissection noted in the proximal segment. There was normalization of the ST segment and immediate restoration of sinus rhythm. There were no thrombi in the perfusion systems or catheters used during the two procedures.

Following this episode, the patient refused surgery. She was treated with tirofiban for 24 h and enoxaparin until discharge. She was gradually weaned from her supports and the rest of her stay was uneventful. In addition to her other medications, she was started on amlodipine for possible coronary vasospasm causing intracoronary thrombosis (ICT). Blood investigations including the thrombophilia profile were normal. During 1-year follow-up, she did not have recurrence of angina but was admitted twice with acute decompensated heart failure and was on optimal anti-heart-failure medications.

## DISCUSSION

We present a patient with ICT in a normal coronary artery following coronary angiography. The exact cause for the thrombus development in this patient is not known. ICT commonly occurs in acute coronary syndrome secondary to plaque erosion or rupture of an atherosclerotic lesion.[1] It may occur spontaneously or as a complication of invasive procedures. ICT with normal coronary arteries may be due to various conditions like embolism, vasospasm, hypercoagulable states, antiphospholipid syndrome, nonatherosclerotic coronary diseases, trauma, an imbalance between oxygen demand and supply, intense sympathetic stimulation, and endothelial dysfunction.[2] Acute closure or myocardial infarction is known to occur in 0.04-0.06% of diagnostic coronary angiography.[3,4] Myocardial infarction is a known complication of coronary angiography, predominantly caused by coronary artery embolization. Embolic events during cardiac catheterization have been attributed to atherosclerotic aortic debris dislodged by catheter manipulation or air embolism. ICT during PCI may occur due to thrombi formed within catheterization instruments or due to guide wires remaining in the arteries for prolonged periods of time. Catheter-induced coronary artery dissection with resultant thrombosis is known during coronary angiography. Invasive catheterization can lead to mechanically induced spasm of coronary arteries which is generally seen next to the catheter tip and is discrete. Vasovagal,[5] contrast-,[6] or drug-eluting stent[7] induced anaphylactoid reactions during catheterization can lead to coronary vasospasm.

In our patient, there was no catheter-induced dissection or vasospasm and no thrombi detected in the devices used during the procedure as well as no vasovagal or anaphylactoid reaction. Spontaneous coronary vasospasm is a frequent cause of acute coronary syndrome and in the recent CASPAR study,[8] it could be documented in nearly 50% of the patients with normal coronaries who were tested by acetylcholine. Coronary vasospasm is reported to occur in 1-5% of PCI and can be induced even solely by guide wire insertion.[9] It is known that prolonged coronary spasm might cause prolonged coronary flow limitation and induce acute thrombus formation without plaque rupture.[10] ICT can occur either as a result of coronary spasm, or it may cause the development of coronary spasm. Another possible cause for ICT could be the rupture of minor plaques with or without spasm that can only be diagnosed using intravascular ultrasound. Our patient demonstrated spontaneous intracoronary thrombosis possibly due to coronary vasospasm that was successfully aspirated using a guide catheter. Previously, thromboaspiration using a guiding catheter has been reported in patients with coronary stenosis and acute coronary syndrome as well as in patients with in-stent thrombosis.[11,12] To our knowledge, this is the first report of thromboaspiration during acute myocardial infarction using a guide catheter in a patient with possible coronary vasospasm and ICT following coronary angiography. Furthermore, this case brings to light a rare and potentially fatal complication of coronary angiography.
